# Supplementary material for: Xenoposeidon is the earliest known rebbachisaurid sauropod dinosaur
Source: PeerJ. 2018 Jul 6;6:e5212. doi: 10.7717/peerj.5212 (PMC6037143; doi:10.7717/peerj.5212)
Supplement: Supplemental Information 1 [file peerj-06-5212-s001.pdf]

Supplementary materials 1. Three-dimensional surface model (11 million polygons) of NHMUK PV R2095, the holotype and only vertebra of *Xenoposeidon proneneukos.* A 3D polygon mesh file was created by Heinrich Mallison (Palaeo3D) in Agisoft Photoscan Pro version 1.3.0 (agisoft.com), from 95 high resolution digital photographs by the author. All 95 images aligned, and resulted in a dense point cloud at maximum resolution of 20,900,043 points and 44,871,128 polygons. Scaling was based on a single 10 cm scale bar created from a high quality scale bar placed in the pictures with the specimen. Available from https://doi.org/10.6084/m9.figshare.5605612.v2 and viewable online at https://sketchfab.com/models/7f88203e0bbb49a194cb254ab05c4b22

Supplementary materials 2. Rotating video, rendered in Rhinoceros 5.0, of three-dimensional surface model (11 million polygons) of NHMUK PV R2095, the holotype and only vertebra of *Xenoposeidon proneneukos.* A 3D polygon mesh file was created by Heinrich Mallison (Palaeo3D) in Agisoft Photoscan Pro version 1.3.0 (agisoft.com), from 95 high resolution digital photographs by the author. All 95 images aligned, and resulted in a dense point cloud at maximum resolution of 20,900,043 points and 44,871,128 polygons. Scaling was based on a single 10 cm scale bar created from a high quality scale bar placed in the pictures with the specimen. Available from https://www.youtube.com/watch?v=2aslY76uUAA
